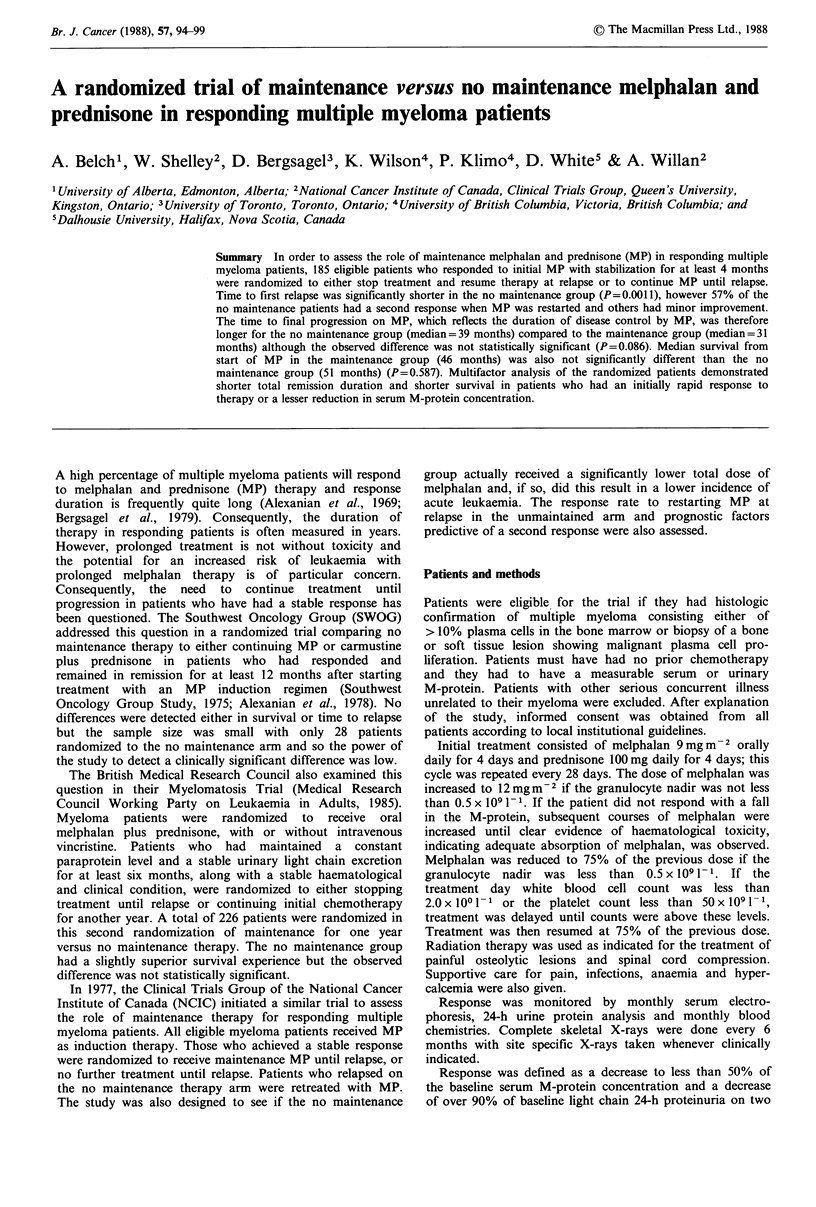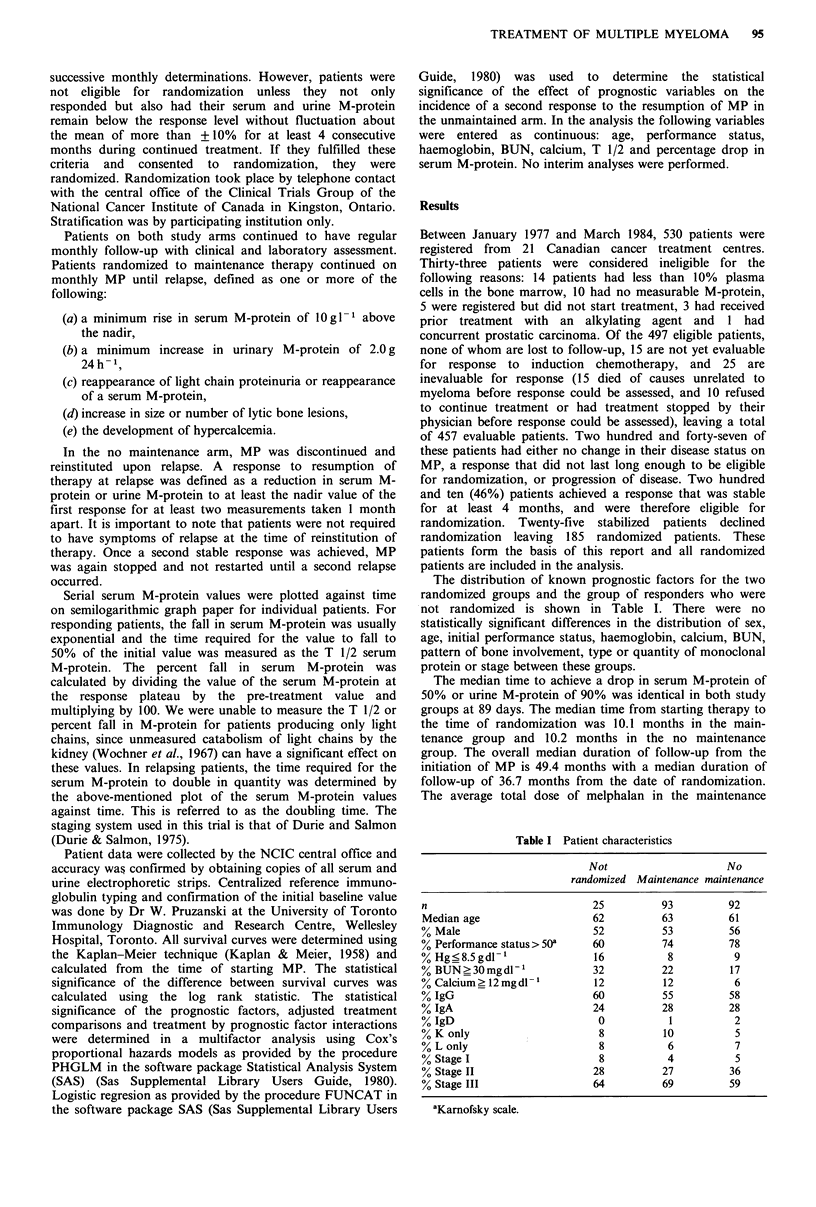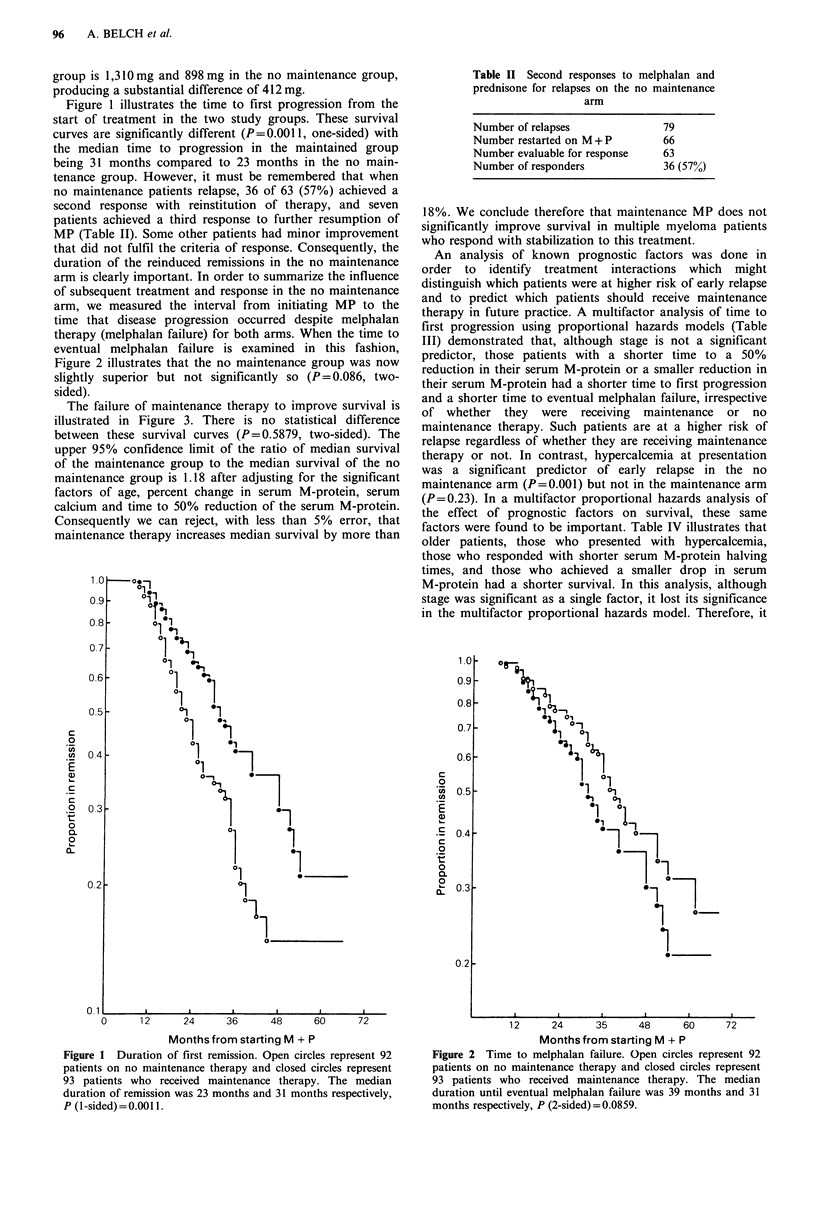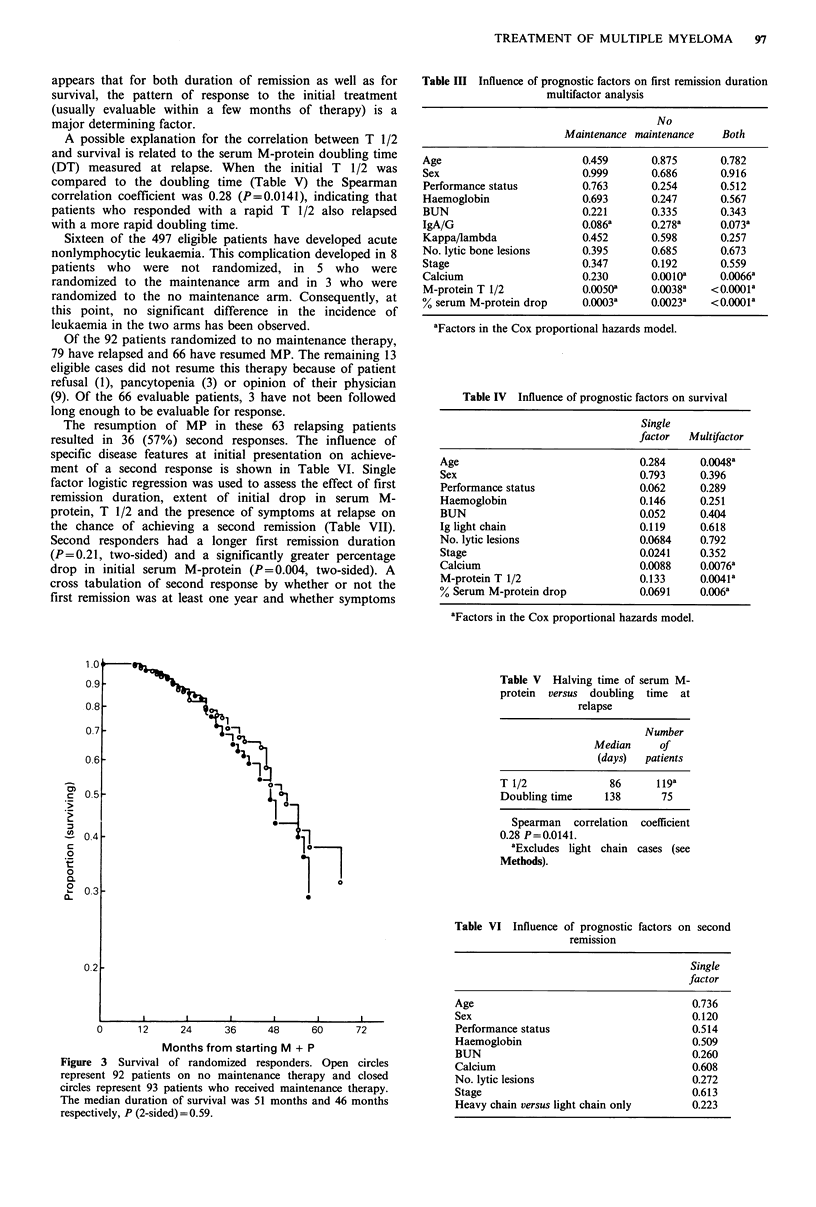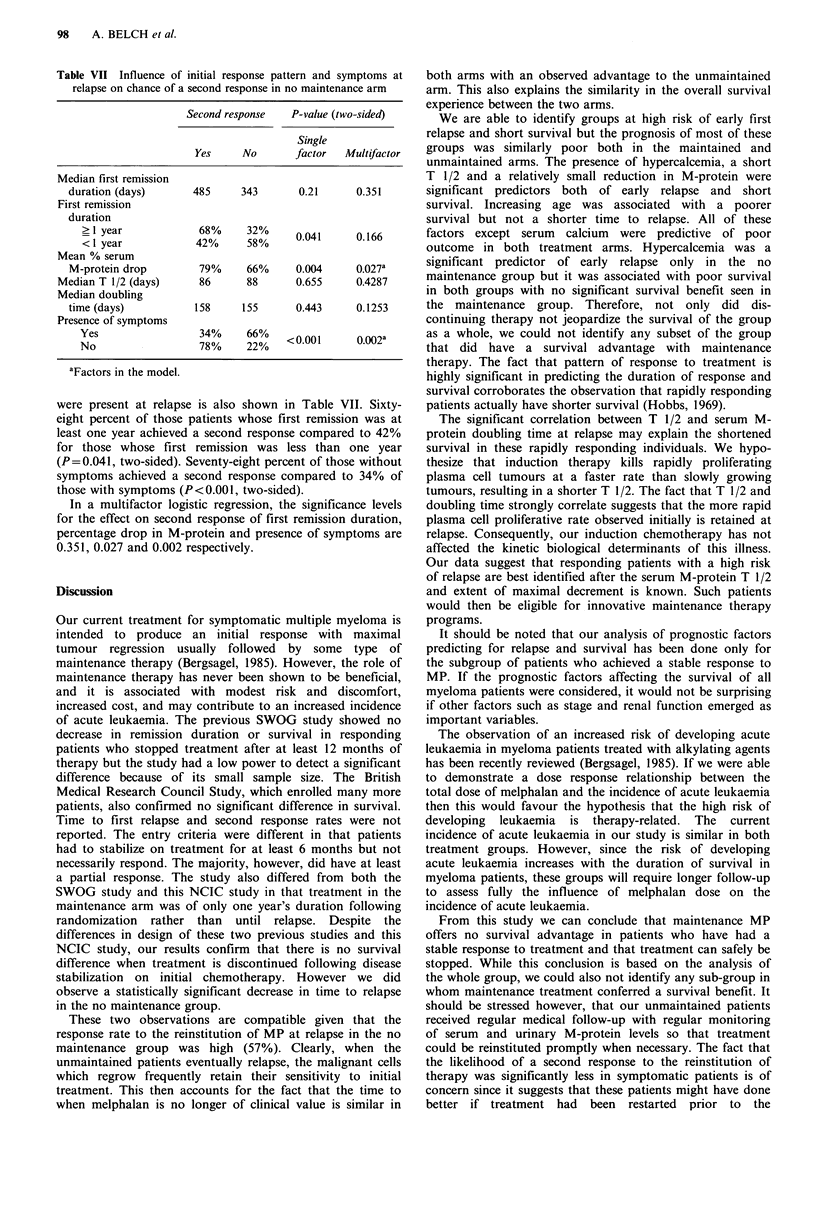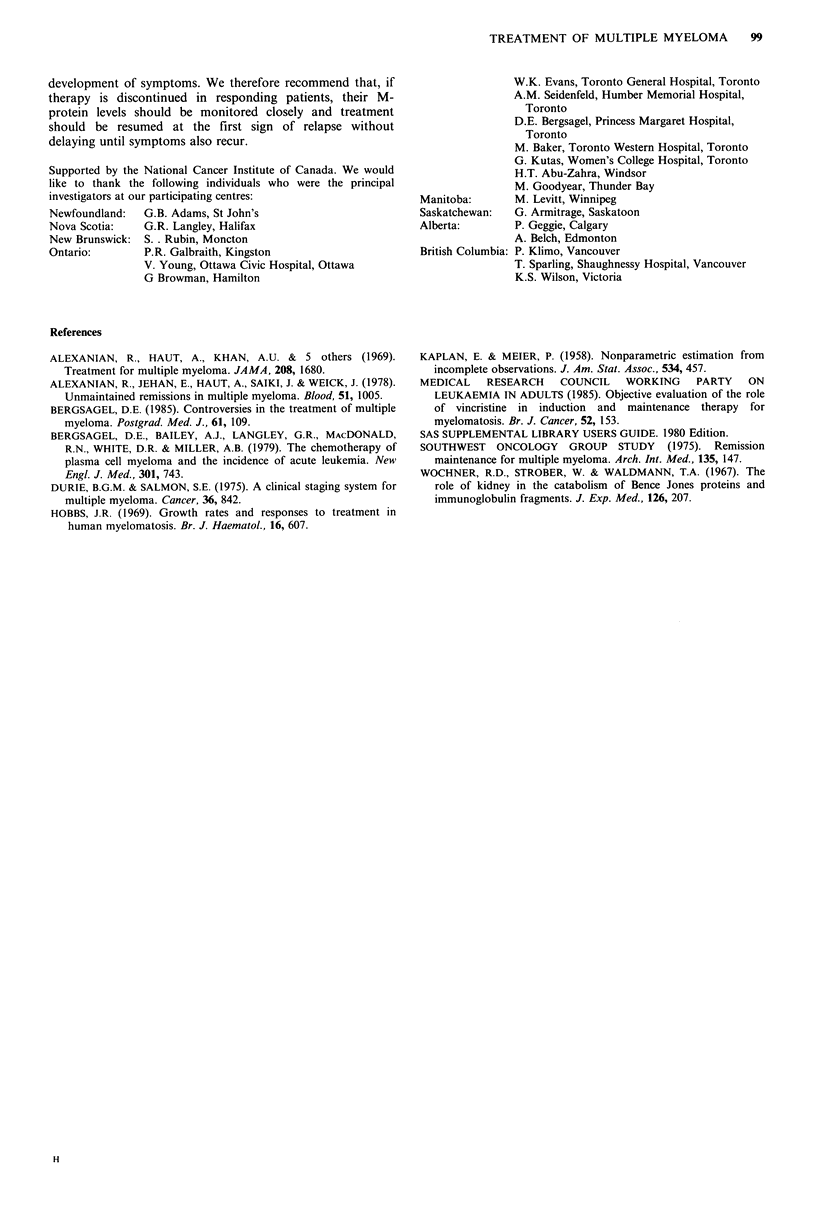# A randomized trial of maintenance versus no maintenance melphalan and prednisone in responding multiple myeloma patients.

**DOI:** 10.1038/bjc.1988.17

**Published:** 1988-01

**Authors:** A. Belch, W. Shelley, D. Bergsagel, K. Wilson, P. Klimo, D. White, A. Willan

**Affiliations:** University of Alberta, Edmonton, Canada.

## Abstract

In order to assess the role of maintenance melphalan and prednisone (MP) in responding multiple myeloma patients, 185 eligible patients who responded to initial MP with stabilization for at least 4 months were randomized to either stop treatment and resume therapy at relapse or to continue MP until relapse. Time to first relapse was significantly shorter in the no maintenance group (P = 0.0011), however 57% of the no maintenance patients had a second response when MP was restarted and others had minor improvement. The time to final progression on MP, which reflects the duration of disease control by MP, was therefore longer for the no maintenance group (median = 39 months) compared to the maintenance group (median = 31 months) although the observed difference was not statistically significant (P = 0.086). Median survival from start of MP in the maintenance group (46 months) was also not significantly different than the no maintenance group (51 months) (P = 0.587). Multifactor analysis of the randomized patients demonstrated shorter total remission duration and shorter survival in patients who had an initially rapid response to therapy or a lesser reduction in serum M-protein concentration.


*Br. J. Cancer* (1988), **57**, 94–99

© The Macmillan Press Ltd., 1988

### A randomized trial of maintenance *versus* no maintenance melphalan and prednisone in responding multiple myeloma patients

A. Belch[1], W. Shelley[2], D. Bergsagel[3], K. Wilson[4], P. Klimo[4], D. White[5] & A. Willan[2]

[1]*University of Alberta, Edmonton, Alberta;* [2]*National Cancer Institute of Canada, Clinical Trials Group, Queen's University, Kingston, Ontario;* [3]*University of Toronto, Toronto, Ontario;* [4]*University of British Columbia, Victoria, British Columbia; and* [5]*Dalhousie University, Halifax, Nova Scotia, Canada*

**Summary** In order to assess the role of maintenance melphalan and prednisone (MP) in responding multiple myeloma patients, 185 eligible patients who responded to initial MP with stabilization for at least 4 months were randomized to either stop treatment and resume therapy at relapse or to continue MP until relapse. Time to first relapse was significantly shorter in the no maintenance group ($P=0.0011$), however 57% of the no maintenance patients had a second response when MP was restarted and others had minor improvement. The time to final progression on MP, which reflects the duration of disease control by MP, was therefore longer for the no maintenance group (median = 39 months) compared to the maintenance group (median = 31 months) although the observed difference was not statistically significant ($P=0.086$). Median survival from start of MP in the maintenance group (46 months) was also not significantly different than the no maintenance group (51 months) ($P=0.587$). Multifactor analysis of the randomized patients demonstrated shorter total remission duration and shorter survival in patients who had an initially rapid response to therapy or a lesser reduction in serum M-protein concentration.

A high percentage of multiple myeloma patients will respond to melphalan and prednisone (MP) therapy and response duration is frequently quite long (Alexanian *et al.*, 1969; Bergsagel *et al.*, 1979). Consequently, the duration of therapy in responding patients is often measured in years. However, prolonged treatment is not without toxicity and the potential for an increased risk of leukaemia with prolonged melphalan therapy is of particular concern. Consequently, the need to continue treatment until progression in patients who have had a stable response has been questioned. The Southwest Oncology Group (SWOG) addressed this question in a randomized trial comparing no maintenance therapy to either continuing MP or carmustine plus prednisone in patients who had responded and remained in remission for at least 12 months after starting treatment with an MP induction regimen (Southwest Oncology Group Study, 1975; Alexanian *et al.*, 1978). No differences were detected either in survival or time to relapse but the sample size was small with only 28 patients randomized to the no maintenance arm and so the power of the study to detect a clinically significant difference was low.

The British Medical Research Council also examined this question in their Myelomatosis Trial (Medical Research Council Working Party on Leukaemia in Adults, 1985). Myeloma patients were randomized to receive oral melphalan plus prednisone, with or without intravenous vincristine. Patients who had maintained a constant paraprotein level and a stable urinary light chain excretion for at least six months, along with a stable haematological and clinical condition, were randomized to either stopping treatment until relapse or continuing initial chemotherapy for another year. A total of 226 patients were randomized in this second randomization of maintenance for one year versus no maintenance therapy. The no maintenance group had a slightly superior survival experience but the observed difference was not statistically significant.

In 1977, the Clinical Trials Group of the National Cancer Institute of Canada (NCIC) initiated a similar trial to assess the role of maintenance therapy for responding multiple myeloma patients. All eligible myeloma patients received MP as induction therapy. Those who achieved a stable response were randomized to receive maintenance MP until relapse, or no further treatment until relapse. Patients who relapsed on the no maintenance therapy arm were retreated with MP. The study was also designed to see if the no maintenance group actually received a significantly lower total dose of melphalan and, if so, did this result in a lower incidence of acute leukaemia. The response rate to restarting MP at relapse in the unmaintained arm and prognostic factors predictive of a second response were also assessed.

### Patients and methods

Patients were eligible for the trial if they had histologic confirmation of multiple myeloma consisting either of >10% plasma cells in the bone marrow or biopsy of a bone or soft tissue lesion showing malignant plasma cell proliferation. Patients must have had no prior chemotherapy and they had to have a measurable serum or urinary M-protein. Patients with other serious concurrent illness unrelated to their myeloma were excluded. After explanation of the study, informed consent was obtained from all patients according to local institutional guidelines.

Initial treatment consisted of melphalan $9\,mg\,m^{-2}$ orally daily for 4 days and prednisone 100 mg daily for 4 days; this cycle was repeated every 28 days. The dose of melphalan was increased to $12\,mg\,m^{-2}$ if the granulocyte nadir was not less than $0.5\times10^{9}\,l^{-1}$. If the patient did not respond with a fall in the M-protein, subsequent courses of melphalan were increased until clear evidence of haematological toxicity, indicating adequate absorption of melphalan, was observed. Melphalan was reduced to 75% of the previous dose if the granulocyte nadir was less than $0.5\times10^{9}\,l^{-1}$. If the treatment day white blood cell count was less than $2.0\times10^{0}\,l^{-1}$ or the platelet count less than $50\times10^{9}\,l^{-1}$, treatment was delayed until counts were above these levels. Treatment was then resumed at 75% of the previous dose. Radiation therapy was used as indicated for the treatment of painful osteolytic lesions and spinal cord compression. Supportive care for pain, infections, anaemia and hypercalcemia were also given.

Response was monitored by monthly serum electrophoresis, 24-h urine protein analysis and monthly blood chemistries. Complete skeletal X-rays were done every 6 months with site specific X-rays taken whenever clinically indicated.

Response was defined as a decrease to less than 50% of the baseline serum M-protein concentration and a decrease of over 90% of baseline light chain 24-h proteinuria on two successive monthly determinations. However, patients were not eligible for randomization unless they not only responded but also had their serum and urine M-protein remain below the response level without fluctuation about the mean of more than ±10% for at least 4 consecutive months during continued treatment. If they fulfilled these criteria and consented to randomization, they were randomized. Randomization took place by telephone contact with the central office of the Clinical Trials Group of the National Cancer Institute of Canada in Kingston, Ontario. Stratification was by participating institution only.

Patients on both study arms continued to have regular monthly follow-up with clinical and laboratory assessment. Patients randomized to maintenance therapy continued on monthly MP until relapse, defined as one or more of the following:

(*a*) a minimum rise in serum M-protein of $10\,g\,l^{-1}$ above the nadir,
(*b*) a minimum increase in urinary M-protein of 2.0 g $24\,h^{-1}$,
(*c*) reappearance of light chain proteinuria or reappearance of a serum M-protein,
(*d*) increase in size or number of lytic bone lesions,
(*e*) the development of hypercalcemia.

In the no maintenance arm, MP was discontinued and reinstituted upon relapse. A response to resumption of therapy at relapse was defined as a reduction in serum M-protein or urine M-protein to at least the nadir value of the first response for at least two measurements taken 1 month apart. It is important to note that patients were not required to have symptoms of relapse at the time of reinstitution of therapy. Once a second stable response was achieved, MP was again stopped and not restarted until a second relapse occurred.

Serial serum M-protein values were plotted against time on semilogarithmic graph paper for individual patients. For responding patients, the fall in serum M-protein was usually exponential and the time required for the value to fall to 50% of the initial value was measured as the T 1/2 serum M-protein. The percent fall in serum M-protein was calculated by dividing the value of the serum M-protein at the response plateau by the pre-treatment value and multiplying by 100. We were unable to measure the T 1/2 or percent fall in M-protein for patients producing only light chains, since unmeasured catabolism of light chains by the kidney (Wochner *et al.*, 1967) can have a significant effect on these values. In relapsing patients, the time required for the serum M-protein to double in quantity was determined by the above-mentioned plot of the serum M-protein values against time. This is referred to as the doubling time. The staging system used in this trial is that of Durie and Salmon (Durie & Salmon, 1975).

Patient data were collected by the NCIC central office and accuracy was confirmed by obtaining copies of all serum and urine electrophoretic strips. Centralized reference immunoglobulin typing and confirmation of the initial baseline value was done by Dr W. Pruzanski at the University of Toronto Immunology Diagnostic and Research Centre, Wellesley Hospital, Toronto. All survival curves were determined using the Kaplan–Meier technique (Kaplan & Meier, 1958) and calculated from the time of starting MP. The statistical significance of the difference between survival curves was calculated using the log rank statistic. The statistical significance of the prognostic factors, adjusted treatment comparisons and treatment by prognostic factor interactions were determined in a multifactor analysis using Cox's proportional hazards models as provided by the procedure PHGLM in the software package Statistical Analysis System (SAS) (Sas Supplemental Library Users Guide, 1980). Logistic regresion as provided by the procedure FUNCAT in the software package SAS (Sas Supplemental Library Users Guide, 1980) was used to determine the statistical significance of the effect of prognostic variables on the incidence of a second response to the resumption of MP in the unmaintained arm. In the analysis the following variables were entered as continuous: age, performance status, haemoglobin, BUN, calcium, T 1/2 and percentage drop in serum M-protein. No interim analyses were performed.

### Results

Between January 1977 and March 1984, 530 patients were registered from 21 Canadian cancer treatment centres. Thirty-three patients were considered ineligible for the following reasons: 14 patients had less than 10% plasma cells in the bone marrow, 10 had no measurable M-protein, 5 were registered but did not start treatment, 3 had received prior treatment with an alkylating agent and 1 had concurrent prostatic carcinoma. Of the 497 eligible patients, none of whom are lost to follow-up, 15 are not yet evaluable for response to induction chemotherapy, and 25 are inevaluable for response (15 died of causes unrelated to myeloma before response could be assessed, and 10 refused to continue treatment or had treatment stopped by their physician before response could be assessed), leaving a total of 457 evaluable patients. Two hundred and forty-seven of these patients had either no change in their disease status on MP, a response that did not last long enough to be eligible for randomization, or progression of disease. Two hundred and ten (46%) patients achieved a response that was stable for at least 4 months, and were therefore eligible for randomization. Twenty-five stabilized patients declined randomization leaving 185 randomized patients. These patients form the basis of this report and all randomized patients are included in the analysis.

The distribution of known prognostic factors for the two randomized groups and the group of responders who were not randomized is shown in Table I. There were no statistically significant differences in the distribution of sex, age, initial performance status, haemoglobin, calcium, BUN, pattern of bone involvement, type or quantity of monoclonal protein or stage between these groups.

The median time to achieve a drop in serum M-protein of 50% or urine M-protein of 90% was identical in both study groups at 89 days. The median time from starting therapy to the time of randomization was 10.1 months in the maintenance group and 10.2 months in the no maintenance group. The overall median duration of follow-up from the initiation of MP is 49.4 months with a median duration of follow-up of 36.7 months from the date of randomization. The average total dose of melphalan in the maintenance

**Table I** Patient characteristics

| | *Not randomized* | *Maintenance* | *No maintenance* |
|---|---|---|---|
| *n* | 25 | 93 | 92 |
| Median age | 62 | 63 | 61 |
| % Male | 52 | 53 | 56 |
| % Performance status > 50[a] | 60 | 74 | 78 |
| % Hg ≦ 8.5 $g\,dl^{-1}$ | 16 | 8 | 9 |
| % BUN ≧ 30 $mg\,dl^{-1}$ | 32 | 22 | 17 |
| % Calcium ≧ 12 $mg\,dl^{-1}$ | 12 | 12 | 6 |
| % IgG | 60 | 55 | 58 |
| % IgA | 24 | 28 | 28 |
| % IgD | 0 | 1 | 2 |
| % K only | 8 | 10 | 5 |
| % L only | 8 | 6 | 7 |
| % Stage I | 8 | 4 | 5 |
| % Stage II | 28 | 27 | 36 |
| % Stage III | 64 | 69 | 59 |

[a]Karnofsky scale.

group is 1,310 mg and 898 mg in the no maintenance group, producing a substantial difference of 412 mg.

Figure 1 illustrates the time to first progression from the start of treatment in the two study groups. These survival curves are significantly different ($P=0.0011$, one-sided) with the median time to progression in the maintained group being 31 months compared to 23 months in the no maintenance group. However, it must be remembered that when no maintenance patients relapse, 36 of 63 (57%) achieved a second response with reinstitution of therapy, and seven patients achieved a third response to further resumption of MP (Table II). Some other patients had minor improvement that did not fulfil the criteria of response. Consequently, the duration of the reinduced remissions in the no maintenance arm is clearly important. In order to summarize the influence of subsequent treatment and response in the no maintenance arm, we measured the interval from initiating MP to the time that disease progression occurred despite melphalan therapy (melphalan failure) for both arms. When the time to eventual melphalan failure is examined in this fashion, Figure 2 illustrates that the no maintenance group was now slightly superior but not significantly so ($P=0.086$, two-sided).

The failure of maintenance therapy to improve survival is illustrated in Figure 3. There is no statistical difference between these survival curves ($P=0.5879$, two-sided). The upper 95% confidence limit of the ratio of median survival of the maintenance group to the median survival of the no maintenance group is 1.18 after adjusting for the significant factors of age, percent change in serum M-protein, serum calcium and time to 50% reduction of the serum M-protein. Consequently we can reject, with less than 5% error, that maintenance therapy increases median survival by more than

**Table II** Second responses to melphalan and prednisone for relapses on the no maintenance arm

| | |
|---|---|
| Number of relapses | 79 |
| Number restarted on M+P | 66 |
| Number evaluable for response | 63 |
| Number of responders | 36 (57%) |

18%. We conclude therefore that maintenance MP does not significantly improve survival in multiple myeloma patients who respond with stabilization to this treatment.

An analysis of known prognostic factors was done in order to identify treatment interactions which might distinguish which patients were at higher risk of early relapse and to predict which patients should receive maintenance therapy in future practice. A multifactor analysis of time to first progression using proportional hazards models (Table III) demonstrated that, although stage is not a significant predictor, those patients with a shorter time to a 50% reduction in their serum M-protein or a smaller reduction in their serum M-protein had a shorter time to first progression and a shorter time to eventual melphalan failure, irrespective of whether they were receiving maintenance or no maintenance therapy. Such patients are at a higher risk of relapse regardless of whether they are receiving maintenance therapy or not. In contrast, hypercalcemia at presentation was a significant predictor of early relapse in the no maintenance arm ($P=0.001$) but not in the maintenance arm ($P=0.23$). In a multifactor proportional hazards analysis of the effect of prognostic factors on survival, these same factors were found to be important. Table IV illustrates that older patients, those who presented with hypercalcemia, those who responded with shorter serum M-protein halving times, and those who achieved a smaller drop in serum M-protein had a shorter survival. In this analysis, although stage was significant as a single factor, it lost its significance in the multifactor proportional hazards model. Therefore, it

**Figure 1** Duration of first remission. Open circles represent 92 patients on no maintenance therapy and closed circles represent 93 patients who received maintenance therapy. The median duration of remission was 23 months and 31 months respectively, $P$ (1-sided) = 0.0011.

**Figure 2** Time to melphalan failure. Open circles represent 92 patients on no maintenance therapy and closed circles represent 93 patients who received maintenance therapy. The median duration until eventual melphalan failure was 39 months and 31 months respectively, $P$ (2-sided) = 0.0859.

appears that for both duration of remission as well as for survival, the pattern of response to the initial treatment (usually evaluable within a few months of therapy) is a major determining factor.

A possible explanation for the correlation between T 1/2 and survival is related to the serum M-protein doubling time (DT) measured at relapse. When the initial T 1/2 was compared to the doubling time (Table V) the Spearman correlation coefficient was 0.28 ($P=0.0141$), indicating that patients who responded with a rapid T 1/2 also relapsed with a more rapid doubling time.

Sixteen of the 497 eligible patients have developed acute nonlymphocytic leukaemia. This complication developed in 8 patients who were not randomized, in 5 who were randomized to the maintenance arm and in 3 who were randomized to the no maintenance arm. Consequently, at this point, no significant difference in the incidence of leukaemia in the two arms has been observed.

Of the 92 patients randomized to no maintenance therapy, 79 have relapsed and 66 have resumed MP. The remaining 13 eligible cases did not resume this therapy because of patient refusal (1), pancytopenia (3) or opinion of their physician (9). Of the 66 evaluable patients, 3 have not been followed long enough to be evaluable for response.

The resumption of MP in these 63 relapsing patients resulted in 36 (57%) second responses. The influence of specific disease features at initial presentation on achievement of a second response is shown in Table VI. Single factor logistic regression was used to assess the effect of first remission duration, extent of initial drop in serum M-protein, T 1/2 and the presence of symptoms at relapse on the chance of achieving a second remission (Table VII). Second responders had a longer first remission duration ($P=0.21$, two-sided) and a significantly greater percentage drop in initial serum M-protein ($P=0.004$, two-sided). A cross tabulation of second response by whether or not the first remission was at least one year and whether symptoms

**Figure 3** Survival of randomized responders. Open circles represent 92 patients on no maintenance therapy and closed circles represent 93 patients who received maintenance therapy. The median duration of survival was 51 months and 46 months respectively, $P$ (2-sided)$=0.59$.

**Table III** Influence of prognostic factors on first remission duration multifactor analysis

| | *Maintenance* | *No maintenance* | *Both* |
|---|---|---|---|
| Age | 0.459 | 0.875 | 0.782 |
| Sex | 0.999 | 0.686 | 0.916 |
| Performance status | 0.763 | 0.254 | 0.512 |
| Haemoglobin | 0.693 | 0.247 | 0.567 |
| BUN | 0.221 | 0.335 | 0.343 |
| IgA/G | 0.086[a] | 0.278[a] | 0.073[a] |
| Kappa/lambda | 0.452 | 0.598 | 0.257 |
| No. lytic bone lesions | 0.395 | 0.685 | 0.673 |
| Stage | 0.347 | 0.192 | 0.559 |
| Calcium | 0.230 | 0.0010[a] | 0.0066[a] |
| M-protein T 1/2 | 0.0050[a] | 0.0038[a] | <0.0001[a] |
| % serum M-protein drop | 0.0003[a] | 0.0023[a] | <0.0001[a] |

[a]Factors in the Cox proportional hazards model.

**Table IV** Influence of prognostic factors on survival

| | *Single factor* | *Multifactor* |
|---|---|---|
| Age | 0.284 | 0.0048[a] |
| Sex | 0.793 | 0.396 |
| Performance status | 0.062 | 0.289 |
| Haemoglobin | 0.146 | 0.251 |
| BUN | 0.052 | 0.404 |
| Ig light chain | 0.119 | 0.618 |
| No. lytic lesions | 0.0684 | 0.792 |
| Stage | 0.0241 | 0.352 |
| Calcium | 0.0088 | 0.0076[a] |
| M-protein T 1/2 | 0.133 | 0.0041[a] |
| % Serum M-protein drop | 0.0691 | 0.006[a] |

[a]Factors in the Cox proportional hazards model.

**Table V** Halving time of serum M-protein *versus* doubling time at relapse

| | *Median (days)* | *Number of patients* |
|---|---|---|
| T 1/2 | 86 | 119[a] |
| Doubling time | 138 | 75 |

Spearman correlation coefficient 0.28 $P=0.0141$.

[a]Excludes light chain cases (see **Methods**).

**Table VI** Influence of prognostic factors on second remission

| | *Single factor* |
|---|---|
| Age | 0.736 |
| Sex | 0.120 |
| Performance status | 0.514 |
| Haemoglobin | 0.509 |
| BUN | 0.260 |
| Calcium | 0.608 |
| No. lytic lesions | 0.272 |
| Stage | 0.613 |
| Heavy chain *versus* light chain only | 0.223 |

**Table VII** Influence of initial response pattern and symptoms at relapse on chance of a second response in no maintenance arm

| | *Second response* | | *P-value (two-sided)* | |
|---|---|---|---|---|
| | *Yes* | *No* | *Single factor* | *Multifactor* |
| Median first remission duration (days) | 485 | 343 | 0.21 | 0.351 |
| First remission duration | | | | |
| ≧1 year | 68% | 32% | 0.041 | 0.166 |
| <1 year | 42% | 58% | | |
| Mean % serum M-protein drop | 79% | 66% | 0.004 | 0.027[a] |
| Median T 1/2 (days) | 86 | 88 | 0.655 | 0.4287 |
| Median doubling time (days) | 158 | 155 | 0.443 | 0.1253 |
| Presence of symptoms | | | | |
| Yes | 34% | 66% | <0.001 | 0.002[a] |
| No | 78% | 22% | | |

[a]Factors in the model.

were present at relapse is also shown in Table VII. Sixty-eight percent of those patients whose first remission was at least one year achieved a second response compared to 42% for those whose first remission was less than one year ($P = 0.041$, two-sided). Seventy-eight percent of those without symptoms achieved a second response compared to 34% of those with symptoms ($P < 0.001$, two-sided).

In a multifactor logistic regression, the significance levels for the effect on second response of first remission duration, percentage drop in M-protein and presence of symptoms are 0.351, 0.027 and 0.002 respectively.

### Discussion

Our current treatment for symptomatic multiple myeloma is intended to produce an initial response with maximal tumour regression usually followed by some type of maintenance therapy (Bergsagel, 1985). However, the role of maintenance therapy has never been shown to be beneficial, and it is associated with modest risk and discomfort, increased cost, and may contribute to an increased incidence of acute leukaemia. The previous SWOG study showed no decrease in remission duration or survival in responding patients who stopped treatment after at least 12 months of therapy but the study had a low power to detect a significant difference because of its small sample size. The British Medical Research Council Study, which enrolled many more patients, also confirmed no significant difference in survival. Time to first relapse and second response rates were not reported. The entry criteria were different in that patients had to stabilize on treatment for at least 6 months but not necessarily respond. The majority, however, did have at least a partial response. The study also differed from both the SWOG study and this NCIC study in that treatment in the maintenance arm was of only one year's duration following randomization rather than until relapse. Despite the differences in design of these two previous studies and this NCIC study, our results confirm that there is no survival difference when treatment is discontinued following disease stabilization on initial chemotherapy. However we did observe a statistically significant decrease in time to relapse in the no maintenance group.

These two observations are compatible given that the response rate to the reinstitution of MP at relapse in the no maintenance group was high (57%). Clearly, when the unmaintained patients eventually relapse, the malignant cells which regrow frequently retain their sensitivity to initial treatment. This then accounts for the fact that the time to when melphalan is no longer of clinical value is similar in both arms with an observed advantage to the unmaintained arm. This also explains the similarity in the overall survival experience between the two arms.

We are able to identify groups at high risk of early first relapse and short survival but the prognosis of most of these groups was similarly poor both in the maintained and unmaintained arms. The presence of hypercalcemia, a short T 1/2 and a relatively small reduction in M-protein were significant predictors both of early relapse and short survival. Increasing age was associated with a poorer survival but not a shorter time to relapse. All of these factors except serum calcium were predictive of poor outcome in both treatment arms. Hypercalcemia was a significant predictor of early relapse only in the no maintenance group but it was associated with poor survival in both groups with no significant survival benefit seen in the maintenance group. Therefore, not only did discontinuing therapy not jeopardize the survival of the group as a whole, we could not identify any subset of the group that did have a survival advantage with maintenance therapy. The fact that pattern of response to treatment is highly significant in predicting the duration of response and survival corroborates the observation that rapidly responding patients actually have shorter survival (Hobbs, 1969).

The significant correlation between T 1/2 and serum M-protein doubling time at relapse may explain the shortened survival in these rapidly responding individuals. We hypothesize that induction therapy kills rapidly proliferating plasma cell tumours at a faster rate than slowly growing tumours, resulting in a shorter T 1/2. The fact that T 1/2 and doubling time strongly correlate suggests that the more rapid plasma cell proliferative rate observed initially is retained at relapse. Consequently, our induction chemotherapy has not affected the kinetic biological determinants of this illness. Our data suggest that responding patients with a high risk of relapse are best identified after the serum M-protein T 1/2 and extent of maximal decrement is known. Such patients would then be eligible for innovative maintenance therapy programs.

It should be noted that our analysis of prognostic factors predicting for relapse and survival has been done only for the subgroup of patients who achieved a stable response to MP. If the prognostic factors affecting the survival of all myeloma patients were considered, it would not be surprising if other factors such as stage and renal function emerged as important variables.

The observation of an increased risk of developing acute leukaemia in myeloma patients treated with alkylating agents has been recently reviewed (Bergsagel, 1985). If we were able to demonstrate a dose response relationship between the total dose of melphalan and the incidence of acute leukaemia then this would favour the hypothesis that the high risk of developing leukaemia is therapy-related. The current incidence of acute leukaemia in our study is similar in both treatment groups. However, since the risk of developing acute leukaemia increases with the duration of survival in myeloma patients, these groups will require longer follow-up to assess fully the influence of melphalan dose on the incidence of acute leukaemia.

From this study we can conclude that maintenance MP offers no survival advantage in patients who have had a stable response to treatment and that treatment can safely be stopped. While this conclusion is based on the analysis of the whole group, we could also not identify any sub-group in whom maintenance treatment conferred a survival benefit. It should be stressed however, that our unmaintained patients received regular medical follow-up with regular monitoring of serum and urinary M-protein levels so that treatment could be reinstituted promptly when necessary. The fact that the likelihood of a second response to the reinstitution of therapy was significantly less in symptomatic patients is of concern since it suggests that these patients might have done better if treatment had been restarted prior to the development of symptoms. We therefore recommend that, if therapy is discontinued in responding patients, their M-protein levels should be monitored closely and treatment should be resumed at the first sign of relapse without delaying until symptoms also recur.

Supported by the National Cancer Institute of Canada. We would like to thank the following individuals who were the principal investigators at our participating centres:

| | |
|---|---|
| Newfoundland: | G.B. Adams, St John's |
| Nova Scotia: | G.R. Langley, Halifax |
| New Brunswick: | S. . Rubin, Moncton |
| Ontario: | P.R. Galbraith, Kingston |
| | V. Young, Ottawa Civic Hospital, Ottawa |
| | G Browman, Hamilton |
| | W.K. Evans, Toronto General Hospital, Toronto |
| | A.M. Seidenfeld, Humber Memorial Hospital, Toronto |
| | D.E. Bergsagel, Princess Margaret Hospital, Toronto |
| | M. Baker, Toronto Western Hospital, Toronto |
| | G. Kutas, Women's College Hospital, Toronto |
| | H.T. Abu-Zahra, Windsor |
| | M. Goodyear, Thunder Bay |
| Manitoba: | M. Levitt, Winnipeg |
| Saskatchewan: | G. Armitrage, Saskatoon |
| Alberta: | P. Geggie, Calgary |
| | A. Belch, Edmonton |
| British Columbia: | P. Klimo, Vancouver |
| | T. Sparling, Shaughnessy Hospital, Vancouver |
| | K.S. Wilson, Victoria |

### References

ALEXANIAN, R., HAUT, A., KHAN, A.U. & 5 others (1969). Treatment for multiple myeloma. *JAMA*, **208**, 1680.

ALEXANIAN, R., JEHAN, E., HAUT, A., SAIKI, J. & WEICK, J. (1978). Unmaintained remissions in multiple myeloma. *Blood*, **51**, 1005.

BERGSAGEL, D.E. (1985). Controversies in the treatment of multiple myeloma. *Postgrad. Med. J.*, **61**, 109.

BERGSAGEL, D.E., BAILEY, A.J., LANGLEY, G.R., MACDONALD, R.N., WHITE, D.R. & MILLER, A.B. (1979). The chemotherapy of plasma cell myeloma and the incidence of acute leukemia. *New Engl. J. Med.*, **301**, 743.

DURIE, B.G.M. & SALMON, S.E. (1975). A clinical staging system for multiple myeloma. *Cancer*, **36**, 842.

HOBBS, J.R. (1969). Growth rates and responses to treatment in human myelomatosis. *Br. J. Haematol.*, **16**, 607.

KAPLAN, E. & MEIER, P. (1958). Nonparametric estimation from incomplete observations. *J. Am. Stat. Assoc.*, **534**, 457.

MEDICAL RESEARCH COUNCIL WORKING PARTY ON LEUKAEMIA IN ADULTS (1985). Objective evaluation of the role of vincristine in induction and maintenance therapy for myelomatosis. *Br. J. Cancer*, **52**, 153.

SAS SUPPLEMENTAL LIBRARY USERS GUIDE. 1980 Edition.

SOUTHWEST ONCOLOGY GROUP STUDY (1975). Remission maintenance for multiple myeloma. *Arch. Int. Med.*, **135**, 147.

WOCHNER, R.D., STROBER, W. & WALDMANN, T.A. (1967). The role of kidney in the catabolism of Bence Jones proteins and immunoglobulin fragments. *J. Exp. Med.*, **126**, 207.